# Effects of curcumin on stem-like cells in human esophageal squamous carcinoma cell lines

**DOI:** 10.1186/1472-6882-12-195

**Published:** 2012-10-24

**Authors:** Taghreed N Almanaa, Michael E Geusz, Roudabeh J Jamasbi

**Affiliations:** 1 Department of Biological Sciences, Bowling Green State University, Bowling Green, OH 43403, USA; 2 Department of Public and Allied Health and Department of Biological Sciences, Bowling Green State University, Bowling Green, OH 43403, USA

**Keywords:** Esophageal cancer, Curcumin, Cancer stem cells, Tumorsphere, ALDH1A1, CD44, NF-κB

## Abstract

**Background:**

Many cancers contain cell subpopulations that display characteristics of stem cells. Because these cancer stem cells (CSCs) appear to provide resistance to chemo-radiation therapy, development of therapeutic agents that target CSCs is essential. Curcumin is a phytochemical agent that is currently used in clinical trials to test its effectiveness against cancer. However, the effect of curcumin on CSCs is not well established. The current study evaluated curcumin-induced cell death in six cancer cell lines derived from human esophageal squamous cell carcinomas. Moreover, these cell lines and the ones established from cells that survived curcumin treatments were characterized.

**Methods:**

Cell loss was assayed after TE-1, TE-8, KY-5, KY-10, YES-1, and YES-2 cells were exposed to 20–80 μM curcumin for 30 hrs. Cell lines surviving 40 or 60 μM curcumin were established from these six original lines. The stem cell markers aldehyde dehydrogenase-1A1 (ALDH1A1) and CD44 as well as NF-κB were used to compare CSC-like subpopulations within and among the original lines as well as the curcumin-surviving lines. YES-2 was tested for tumorsphere-forming capabilities. Finally, the surviving lines were treated with 40 and 60 μM curcumin to determine whether their sensitivity was different from the original lines.

**Results:**

The cell loss after curcumin treatment increased in a dose-dependent manner in all cell lines. The percentage of cells remaining after 60 μM curcumin treatment varied from 10.9% to 36.3% across the six lines. The cell lines were heterogeneous with respect to ALDH1A1, NF-κB and CD44 expression. KY-5 and YES-1 were the least sensitive and had the highest number of stem-like cells whereas TE-1 had the lowest. The curcumin-surviving lines showed a significant loss in the high staining ALDH1A1 and CD44 cell populations. Tumorspheres formed from YES-2 but were small and rare in the YES-2 surviving line. The curcumin-surviving lines showed a small but significant decrease in sensitivity to curcumin when compared with the original lines.

**Conclusion:**

Our results suggest that curcumin not only eliminates cancer cells but also targets CSCs. Therefore, curcumin may be an effective compound for treating esophageal and possibly other cancers in which CSCs can cause tumor recurrence.

## Background

Esophageal cancer is the eighth most common cancer worldwide and the sixth most common cause of death among cancers [1]. Of the two types of esophageal cancer, adenocarcinoma (EAC) and squamous cell carcinoma (ESCC), 90% are ESCC, with rates increasing significantly in developing countries [2]. About 13% of ESCC patients survive for five years after diagnosis [3]. The prognosis of ESCC is often poor due to lack of effective treatment [4]. As a result of this limitation, newer agents and novel approaches are imperative. Of particular interest is the chemotherapeutic application of curcumin, the major active ingredient of turmeric (*Curcumin longa*) [5-8]. Curcumin induces cell death in some cancers, such as gastric and colon cancers [9], human melanoma [10], and lung cancer [11] without major cytotoxic effects on healthy cells [12,13]. Curcumin induces cell death through a variety of mechanisms by targeting pathways acting through a range of transcription factors, membrane receptors, kinases, and cytokines (reviewed by Anand et al. [14]). Therefore, curcumin has a potential treatment value for cancer either alone [15] or in combination with other treatments, namely chemotherapy [16] and radiation treatments [17]. Although it is rapidly degraded and thus may have little effect outside of the digestive tract [18,19], curcumin could be effective for treating ESCC because of its direct contact with epithelial cells lining the esophagus during ingestion.

One possible reason for the poor prognosis of ESCC is the presence of cancer stem cells (CSCs) in the tumor [20,21]. It is believed that CSCs regenerate themselves and differentiate into non-CSCs that constitute most of the tumor volume [22-25]. Furthermore, CSCs tend to resist currently used cancer treatments, specifically chemotherapy and radiation therapy [26-28]. Therefore, the development of effective treatments for cancer should target this cell subpopulation [29]. Interestingly, curcumin with or without 5-fluorouracil and oxaliplatin significantly reduced the number of cells showing CSC-markers CD44 and CD166 in a colon cancer cell line that had survived previous treatment with 5-fluorouracil [30]. More recently, Nautiyal et al. [31] reported that a combination of curcumin and the chemotherapeutic agent dasatinib eliminates mRNA stem cell markers—specifically, ALDH, CD44, CD133, and CD166—that are enriched in the chemo-resistant colon cancer cells. Similarly, Fong et al. [32] reported that curcumin decreased the side-population associated with stem cell populations in the C6 glioma cell line, as determined by negative Hoechst 33342 nuclear staining.

CSCs have high expression of aldehyde dehydrogenase (ALDH) that can be used as a potential marker for identifying and isolating CSCs [33-35]. ALDH is a detoxifying enzyme responsible for the oxidation of both intracellular aldehydes and xenobiotic aldehydes such as cyclophosphamide [36,37]. High ALDH expression could also indicate the aggressiveness, invasiveness, or metastatic capability associated with different cancers [27,38]. ALDH1A1 is a member of the ALDH family that participates in alcohol metabolism and offers cellular protection against cytotoxic drugs [39]. Although immunohistochemistry with a specific antibody against ALDH1A1 has been used to identify both somatic stem cells and cancer stem cells [38,40,41], ALDH1A1 expression has not been evaluated in human esophageal cancer.

Another marker for CSCs is CD44 (cluster of differentiation 44). CD44 is an integral cell membrane glycoprotein involved in cell-cell interaction [42]. CD44 has been identified in many types of CSCs, including breast cancer [43], head and neck cancer [44], gastric cancer [45] as well as ESCC [46]. High CD44 expression is also associated with metastatic and invasive capabilities [47].

An additional property of cancer stem cells (CSCs) is the ability to grow as non-adherent spheres when cultured in serum-free medium supplemented with basic fibroblast growth factor (bFGF) and epidermal growth factor (EGF) [48-50]. Recently, non-adherent sphere cultures have been used as an effective approach to enrich and identify stem cells or CSCs [51,52].

In this study, curcumin-surviving cell lines were established from six human ESCC lines. The CSC markers ALDH1A1 and CD44, curcumin toxicity, and tumorsphere formation were examined. When the surviving cell lines were compared with the original untreated lines, the results indicated that curcumin produced a permanent reduction in the CSC-like subpopulation of these cells.

## Methods

### Cell lines and culture conditions

The six human esophageal squamous cancer cell lines (TE-1, TE-8, KY-5, KY-10, YES-1, and YES-2) were obtained from various sources [53-56]. The cell lines were cultured in Dulbecco's modified eagle medium (DMEM) supplemented with penicillin (100 units/ml), streptomycin (100 μg/ml), L-glutamine (2 mM) (GIBCO, Invitrogen, NY), and 10% fetal bovine serum (FBS, Summit Biotechnology, Ft. Collins, CO). This medium was designated as complete DMEM. The cells in culture were maintained in 100-mm tissue culture dishes (Falcon, Lincoln Park, NJ) at 37°C in a humidified incubator containing 5% CO_2_. When the cells reached near confluency, they were washed with phosphate-buffered saline (PBS) once and incubated with trypsin (Trypsin-EDTA 0.25%, GIBCO, Invitrogen, NY) for two min. After incubation, the cells were washed once with complete DMEM to inactivate trypsin. The cells were then collected by centrifugation at 800 x g, resuspended in complete DMEM and plated into new culture dishes.

### Morphological examination

To examine the morphology of the ESCC cell lines, each cultured line was trypsinized, washed and counted using a hemocytometer (Hausser Scientific, PA). The cells were then plated into 100-mm dishes (1×10^5^ cells/plate) in complete DMEM and incubated at 37°C. When the cells reached near confluency, approximately 70-75%, the cultures were photographed using a phase-contrast microscope (Olympus CK40) equipped with a color digital camera (Kodak M575).

### Curcumin treatment

To determine the effect of different doses of curcumin on the ESCC lines, each cell line was plated in 24-well plates (Corning) at a density of 5×10^4^ cells/well in complete DMEM, and incubated at 37°C. Curcumin (Sigma-Aldrich, St Louis, MO) was dissolved in dimethyl sulfoxide (DMSO) to make a stock solution of 20 mM curcumin. For each experiment, stock solution was diluted to the final concentrations (20, 30, 40, 60 and 80 μM) in complete DMEM. Twenty-four hours after plating, the wells were washed with PBS once, and 1 ml of each curcumin dosage was added (4 wells/dosage) and incubated for 30 hrs. Four other wells received complete DMEM or complete DMEM with 0.2% DMSO as controls. The inhibition of cell growth in response to curcumin treatment was assayed with the crystal violet staining assay according to a standard protocol [57]. Briefly, the cells in each well were washed once with PBS, stained with 200 μl crystal violet in 50% ethanol (10 mg/ml), and incubated at room temperature for 10 min. The crystal violet was then removed and the wells were washed two times with distilled water, and 600 μl of 1% sodium dodecylsulfate (SDS) in water was added to each well. The plates were maintained overnight at room temperature on a shaker to dissolve the dye. Absorbance was read at 490 nm using a Wallac Victor 1420 Multilabel plate reader (Perkin Elmer, Waltham, MA). The background readings from the SDS solution were subtracted from each reading and the percentage of cells remaining after curcumin treatment was calculated.

### Curcumin-surviving subpopulation

To select for a curcumin-surviving cell subpopulation, each of the ESCC cell lines was cultured in 100-mm dishes (1×10^5^ cells/dish) with complete DMEM and incubated at 37°C until reaching near confluency, approximately 80%. After incubation, the medium was removed and the cells were incubated in complete DMEM containing either 40 or 60 μM curcumin dissolved in 0.2% DMSO for 30 hrs. After incubation, the medium-containing curcumin was removed and replaced with complete DMEM to allow cells that survived the treatment to grow. After the surviving cells formed colonies (normally four weeks after treatment), the colonies were counted, trypsinized, and passaged. The cell lines that developed were designated the curcumin-surviving cell lines.

### Exposure of the curcumin-surviving cell lines to curcumin

To compare the effect of curcumin on the original cell lines and the curcumin-surviving cell lines, the curcumin-surviving lines were cultured in 24-well plates (5×10^4^ cells/ well) in complete DMEM and incubated at 37°C. Twenty-four hours after plating, wells were treated with 40 or 60 μM curcumin and incubated for 30 hrs (4 wells/dosage). The inhibition of cell growth in response to curcumin treatment was assayed with the crystal violet staining assay as described above.

### Immunocytochemistry

To assess the expression of the stem cell markers ALDH1A1 and CD44 in both the original ESCC cell lines and the curcumin-surviving cell lines, each line was grown on glass cover slips in complete DMEM. After two days, the cover slips were rinsed with PBS and fixed with methanol for 5 min. The cover slips were then washed three times with PBS, exposed to 0.3% hydrogen peroxide for 3 min, and rinsed three times with PBS. Next, the cells were blocked with normal goat serum (Rockland, Gilbertsville, PA) at 1:100 dilution in PBS for 30 min. The cover slips were then incubated with a primary antibody for 2 hrs at room temperature, after which the cover slips were washed three times with PBS and incubated with a secondary antibody for 30 min. For ALDH1A1 staining, a rabbit anti-human ALDH1A1 antibody (Proteintech Group, Chicago, IL) at 1:100 dilution and a goat anti-rabbit antibody conjugated with horseradish peroxidase (Rockland, Gilbertsville, PA) at 1:1000 dilution were used. For CD44 staining, a mouse monoclonal anti-human CD44 antibody (BD Biosciences, Franklin Lakes, NJ) at 1:10 dilution and a goat anti-mouse horseradish peroxidase-conjugated antibody (Sigma-Aldrich, St Louis, MO) at 1:40 dilution were used. The cells were then reacted with ImmunPACT DAB (Vector Laboratories, Burlingame, CA) for 10 min. The cover slips were washed three times with water, dehydrated in an ethanol series, cleared with Citrosolve (Fisher, Pittsburgh, PA), and mounted on glass slides with Permount (Fisher, Pittsburgh, PA). Control coverslips did not receive the primary antibody but were otherwise treated identically.

### Image analysis

The images of cell cultures immunostained for ALDH1A1 or CD44 were analyzed with ImageJ (NIH) and OriginLab (OriginLab) software. To determine the intensity of staining, all cells were imaged with a 12-bit digital camera (MicroMax, Princeton Instruments) and a Zeiss Axiophot microscope. The images were thresholded identically using a pixel intensity (brightness) value of 500 analog-to-digital units (ADUs). For counting cells, ImageJ was used to identify cells within a normal size range (500–4000 pixels in area) that did not touch the edge of the image.

### Enzyme-linked immunosorbent assay (ELISA)

The level of RelA (p65), a subunit of transcription factor NF-κB, and ALDH1A1 was examined in both curcumin-surviving cells and original cell lines. ELISA was performed as described previously [58]. Briefly, each cell line was cultured in 96-well plates (5×10^4^ cells/well) and incubated for 24 hrs at 37°C. After incubation, the wells were washed once with PBS, fixed with methanol for 5 min, and then washed twice with PBS. Anti-p65 at 1:50 dilution (Santa Cruz Biotechnology, SC-109, 200 μg/ml) and anti-ALDH1A1 at 1:500 dilution (Proteintech Group, 15910-1-AP, 133 μg/ml) were prepared in complete DMEM. The wells were incubated with 100 μl of each antibody (3 wells/antibody dilution) for 2 hrs at 37°C. After incubation, the wells were washed with PBS three times and incubated with 100 μl/well of β-galactosidase-conjugated goat anti-rabbit secondary antibody (Southern Biotech, Birmingham, AL, 1:500 dilution in complete DMEM) for 2 hrs at 37°C. After incubation, the wells were washed with PBS three times and 100 μl/well of substrate solution (1 mg/ml p-nitrophenyl-β-D-galactopyranoside in phosphate buffer, pH 8.0) was added and incubated at 37°C for one hour in darkness. The development of yellow color indicated a positive reaction. The antigen-antibody reactions were measured by determining the optical density (O.D.) at 410 nm using a microwell plate reader (MR-600, Dynatech Lab Inc.).

### Tumorsphere formation

To examine the ability of the cell line to form spheres, YES-2 and YES-2S cell lines were cultured in stem cell medium. Cells were maintained as a monolayer in complete DMEM until they were nearly confluent. Cells were trypsinized, collected, and washed two times to remove serum, then suspended in serum-free DMEM/F12 supplemented with penicillin (100 units/ml), streptomycin (100 μg/ml), 20 ng/ml human epidermal growth factor (EGF), 20 ng/ml human basic fibroblast growth factor (bFGF), and B27 supplement (Invitrogen, Carlsbad, CA, USA). The cells were subsequently cultured in ultra-low attachment 60-mm plates (Corning Inc., Corning, NY, USA) at a density of 10.000 cells/ plate. Plates were incubated at 37°C in a humidified 5% CO_2_ incubator for a week and then formation of free-floating spheres was evaluated. The experiment was performed in triplicate.

### Statistical analysis

For statistical analysis OriginPro 7.5 (OriginLab) was used. Statistical comparisons between two groups were made using Student’s *t*-test. For comparing more than two groups, one-way analysis of variance (ANOVA) was used followed by the Scheffe multiple comparisons test with a significance of p< 0.05. All errors are shown as standard deviation (SD).

## Results

### Morphological characteristics of the ESCC lines

**Figure 1:**
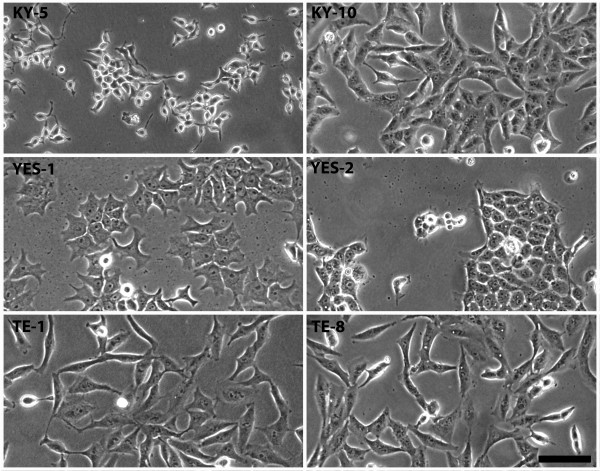
**Morphology of the six ESCC cell lines. **Each cell line had a unique cell shape and size as shown by phase contrast microscopy. Cell morphology agreed with published descriptions [53-56]. Scale bar equals 100 μm.

The characteristics of patients, tumors, and esophageal cell lines derived from each tumor are listed in Table 1. All cell lines grew as adherent monolayers with unique morphological characteristics in size and shape as shown in Figure 1. The morphological characteristics of the ESCC cell lines were similar to the morphologies that were originally described [53-56].

**Table 1:** Reported characteristics of cell lines derived from esophageal squamous cell carcinomas

**Cell line**	**Patient information**		**Cell line properties**			**References**
	**Age**	**Tumor differentiation**	**Cell doubling time (hrs)**	**Tumorigenicity**	**Morphology**	
KY-5 (KYSE-50)	58	Poor	28.2	Yes	Growing as a cluster with smaller cells in the center	[53]
KY-10 (KYSE-110)	63	Poor	19.1	Yes	Spindle shape	[53]
YES-1	50	Poor	35.2	Yes	Polygonal to spindle shape	[54]
YES-2	81	Moderate	23.7	Yes	Polygonal epithelial shape	[55]
TE-1	58	Well	27.2*	Yes	Spindle shape	[56]
TE-8	63	Moderate	25.7*	N.A.	Spindle shape	[56]

All tumors were from male patients. Cell line tumorigenicity was determined by subcutaneous injection into nude mice. N.A. = not available. *Doubling time was determined in the present study.

### Cytotoxic effect of curcumin on the ESCC lines

**Figure 2:**
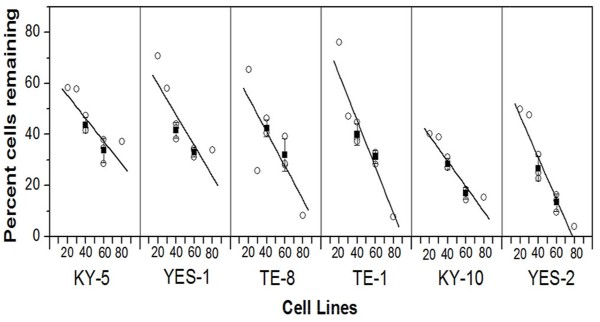
**Effects of curcumin on six ESCC cell lines. **At 60 μM curcumin, YES-2 and KY-10 were more sensitive than KY-5, YES-1, TE-8, and TE-1 (p<0.05, ANOVA, F=16.44, p<0.0001). Cell loss was measured by a standard crystal violet assay after incubation with 20 to 80 μM curcumin for 30 hrs. Each point represents the effect of one curcumin dosage (20, 30, 40, 60 or 80 μM). For 40 and 60 μM the error bar indicates mean (±SD) of three experiments.

To assess the sensitivity of the six human ESCC lines to curcumin, the cell lines (TE-1, TE-8, KY-5, KY-10, YES-1, and YES-2) were plated in 24-well plates and maintained in culture for 24 hrs and then exposed to 20, 30, 40, 60, and 80 μM curcumin and to 0.2% DMSO as a control for 30 hrs. The resulting cell density was determined by a standard crystal violet assay. The cell density remaining after the treatment is shown in the dose–response curves (Figure 2). Curcumin caused a reduction in cells in a dosage-dependent manner in all six ESCC cell lines. However, KY-5 had the highest percentage of cells remaining at the highest curcumin concentration, and the YES-2 cell line had the lowest percentage of cells remaining. When the cell lines were ranked from low to high according to their sensitivity to 60 μM curcumin the order was: KY-5, YES-1, TE-8, TE-1, KY-10, and YES-2. The range of the percentage of cells remaining after the curcumin treatment across the six cell lines was 10.9% to 36.3% at 60 μM curcumin. At 60 μM curcumin, the YES-2 and KY-10 were more sensitive than the KY-5, YES-1, TE-8, and TE-1 (p<0.05 according to one-way ANOVA, F=16.44, p<0.0001).

### Comparison of the ESCC lines and the curcumin-surviving lines

According to the dose–response results, 60 μM curcumin was chosen to select for the curcumin-surviving cell line subpopulations, indicated by “S”. To establish this subpopulation, 1×10^5^ cells from each ESCC line were plated into 100-mm tissue culture dishes and incubated until they reached near confluency. The cells were then exposed to 60 μM curcumin for 30 hrs, and the surviving colonies formed were counted. The number of colonies formed after 60 μM curcumin treatment varied among the six cell lines. YES-2 cell line did not form any colony at the concentration of 60 μM curcumin, therefore it was treated with a lower concentration (40 μM) curcumin. The number of surviving colonies formed after 60 μM curcumin was 0, 8, 17, 19, 25, and too many colonies to count for YES-2, TE-1, TE-8, KY-10, YES-1, and KY-5, respectively. The KY-5 cell line had the highest number of surviving colonies, whereas the YES-2 cell line did not form any colonies after 60 μM curcumin but formed 15 colonies after treatment with 40 μM curcumin.

To further distinguish between the original ESCC cell lines and the curcumin-surviving lines, we measured NF-κB, ALDH1A1, and CD44 levels. We also examined the effect of curcumin on these lines and their ability to form tumorspheres.

### Evaluation of ALDH1A1 and NF-κB levels using ELISA

**Figure 3:**
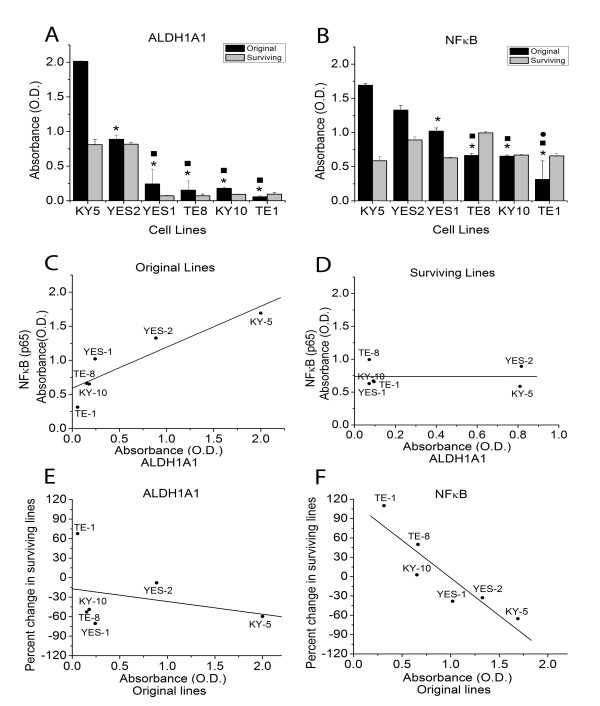
**NF-κB and ALDH1A1 levels in original ESCC cell lines and curcumin-surviving lines measured using ELISA. **(**A**) ALDH1A1 expression levels varied between the six original ESCC lines. ALDH1A1 expression was significantly higher in KY-5 than in all other lines (asterisk). YES-2 had higher expression than the other four lines (filled square). (**B**) NF-κB levels also varied between the cell lines. KY-5 showed significantly higher NF-κB levels than the other four lines (asterisk). YES-2 also had higher NF-κB levels than the other three cell lines (filled square). YES-1 was higher than one line (filled circle). All significant differences were based on one-way ANOVA (F=152.71, p<0.0001 for ALDH1A1 and F=55.73, p<0.0001 for NF-κB). (**C**) ALDH1A1 and NF-κB levels in the original cell lines showed a strong correlation (R=0.903, linear regression, p=0.0134). (**D**) This relationship was absent in the surviving lines (R=−0.004, p=0.994). (**E**) All cell lines but one (TE-1) had lower ALDH1A1 levels in the surviving lines than in the original lines. This decline was not a function of ALDH1A1 levels in the original lines (R=−0.285, p=0.583). (**F**) In contrast, the change in NF-κB levels between original and surviving lines was inversely correlated with NF-κB levels in the original lines (R=−0.901, p=0.0139). The average optical density at 410 nm of three wells (±SD) is shown. Curcumin-surviving cell lines were derived from cells that survived 60 μM curcumin treatment, except YES-2S, which survived 40 but not 60 μM curcumin.

The ALDH1A1 and NF-κB expression levels were measured in the original ESCC lines and the curcumin-surviving lines using ELISA. As shown in Figure 3A and 3B, the levels of ALDH1A1 and NF-κB showed significant variation among the six original ESCC lines. ALDH1A1 expression level was significantly higher in KY-5 than in all other lines. YES-2 had higher ALDH1A1 expression than YES-1, TE-8, KY-10 and TE-1. Also, KY-5 showed significantly higher NF-κB levels than YES-1, TE-8, KY-10 and TE-1. YES-2 had higher NF-κB expression than TE-8, KY-10 and TE-1, and YES-1 was higher than TE-1. All significant differences were based on one-way ANOVA (F=152.71, p<0.0001 for ALDH1A1 and F=55.73, p<0.0001 for NF-κB). In the original cell lines, ALDH1A1 expression was positively correlated with NF-κB expression as shown in Figure 3C (R=0.903, linear regression, p=0.0134). The KY-5 cell line showed the highest levels of ALDH1A1 and NF-κB, whereas the TE-1 cell line showed the lowest levels of both markers. However, there was no relationship between ALDH1A1 and NF-κB expression in the surviving lines as shown in Figure 3D (R= −0.004, p=0.994).

The results also showed that the curcumin-surviving lines had lower ALDH1A1 levels when compared with the original cell lines, except for TE-1 (Figure 3A). This decline was not a function of ALDH1A1 levels in the original lines as shown in Figure 3E (R=−0.285, p=0.583). In addition, the results also showed that KY-5S, YES-1S and YES-2S had lower NF-κB levels when compared with the original cell lines (Figure 3B). However, TE-1S and TE-8S showed increased NF-κB levels relative to the original lines, but KY-10S showed no difference from KY-10. As shown in Figure 3F, the change in NF-κB levels between original and surviving lines was inversely correlated with NF-κB levels in the original lines (R= −0.901, p=0.0139).

### Assessment of ALDH1A1 expression in ESCC and curcumin-surviving lines by immunocytochemistry

**Figure 4:**
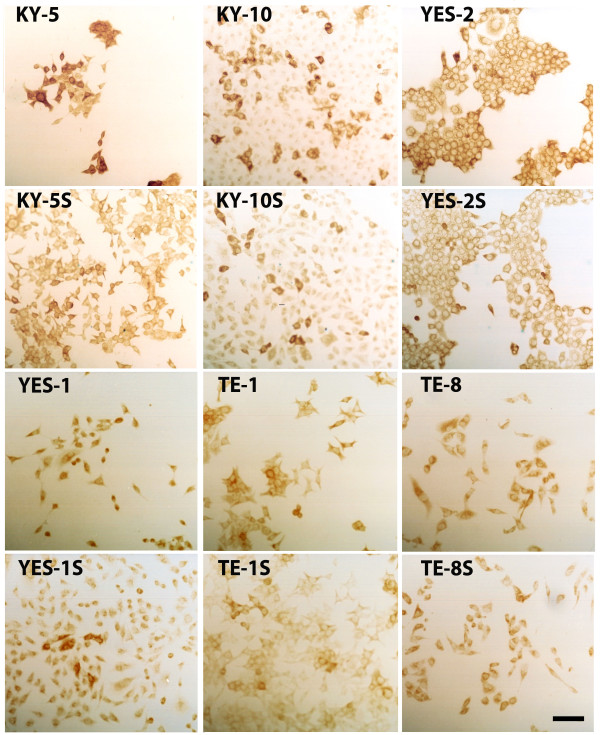
**ALDH1A1 immunocytochemistry. **After immunostaining for ALDH1A1, all six ESCC lines and curcumin-surviving lines showed distinct high and low-staining cells. Cells were stained 48 hrs after plating and then staining intensity of individual cells was quantified. **“S”** indicates curcumin-surviving cell lines. Scale bar = 100 μm.

ALDH1A1 immunostaining was examined in the six original ESCC lines by measuring the staining intensity of individual cells. As shown in Figure 4, high ALDH1A1 staining was detected in the cytoplasm of the six cell lines and this was significantly different among the lines according to mean intensity (F=307, p<0.0001). Staining for ALDH1 was significantly higher in KY5 than in all other lines. YES-2 had significantly higher staining than YES-1, TE-8, TE-1, and KY-10. Also, TE-8 staining was significantly higher than YES-1, TE-1 and KY-10.

**Figure 5:**
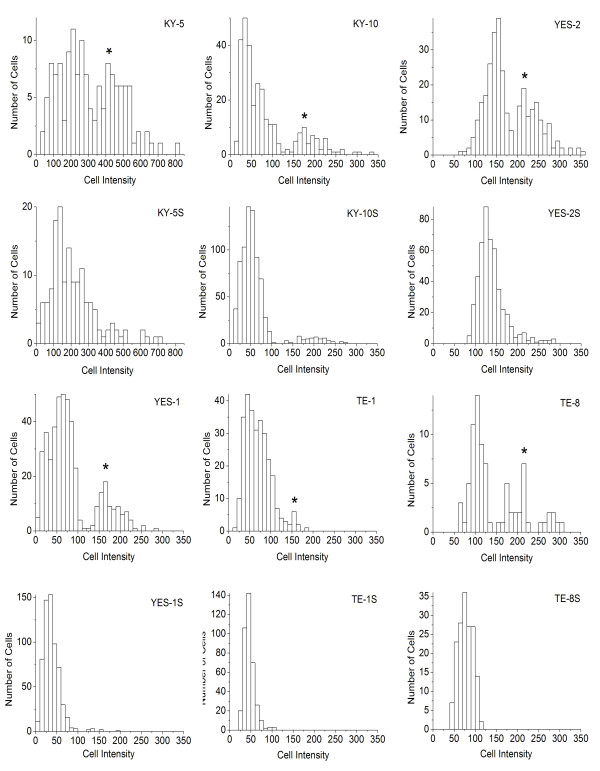
**ALDH1A1 immunostaining of original ESCC cell lines and curcumin-surviving lines. **The histograms summarize cell counts according to staining intensity as shown in Figure. 4. The maxima of the high-staining subpopulations in the original cell lines are shown by the asterisk. Curcumin-surviving cell lines, indicated with “**S**”, showed a significant loss of the high-staining subpopulation. The mean intensity of ALDH1A1 expression was significantly different among the original lines (F=307.047, p<0.0001). KY-5 staining was significantly higher than all other lines. YES-2 was significantly higher than YES-1, TE-8, TE-1, and KY-10. Also, TE-8 was significantly higher than YES-1, TE-1 and KY-10. The mean intensity of ALDH1A1 expression levels was also significantly different among the surviving lines (F=611.440, p<0.0001). KY-5S staining was significantly higher than all other surviving lines. YES-2S was significantly higher than TE-8S, KY-10S, TE-1S and YES-1S. TE-8S was higher than KY-10S, YES-1S and TE-1S. Also, KY-10S was significantly higher than YES-1S and TE-1S. The mean intensity of high-staining cells in the original cell lines was 477.80 ± 93.82, 243.77 ± 33.54, 222.23 ± 42.02, 199.70 ± 39.31, 180.88 ± 28.31, and 149.67 ± 16.29 for KY-5, YES-2, TE-8, KY-10, YES-1, and TE-1, respectively. The mean intensity of high-staining cells in the surviving cell lines was 496.93 ±108.38, 234.95 ±28.23, 206.67 ±26.77, and 145.55 ± 24.52 for KY-5S, YES-2S, KY-10S, and YES-1S.

Within all of the six original lines, there were two subpopulations, each with its own distinctive peak in the histogram, (high and low ALDH1A1-staining subpopulations), as shown in Figure 5. The high and low subpopulations were separated by the minimum between the two peaks. Mean staining intensity was significantly different between the two subpopulations (F=941, p<0.0001). The mean intensity of the high-staining subpopulation was about twice the mean of the low-intensity cells. For example, the mean of the low intensity population in KY-5 was 192.56 ± 80.48, and the mean of the high intensity was 477.80 ± 93.82. When arranged by mean intensity of the high-staining subpopulation, the cell lines were ranked: KY-5 (477.80 ± 93.82), YES-2 (243.77 ± 33.54), TE-8 (222.23 ± 42.02), KY-10 (199.70 ± 39.31), YES-1 (180.88 ± 28.31), and TE-1 (149.67 ± 16.29). When the percentage of cells in the high-staining subpopulation was analyzed, KY-5 showed the greatest percentage and TE-1 the lowest. The percentage of cells with high ALDH1A1 staining ranged from 5.6% to 40.4%.

Like the original cell lines, there was a significant high-staining subpopulation within each surviving line except TE-1S and TE-8S, as shown in Figures 4 and 5 (F=1400.88, p<0.0001). As in the original lines, the high-staining subpopulation mean intensity was about twice that of the low staining subpopulation. For example, the mean of the low staining subpopulation in KY-5S was 172.59 ± 83.55 and the high was 496.93 ± 108.38. When arranged by mean intensity of the high-staining subpopulation, the cell lines were ranked: KY-5S (496.93 ±108.37), YES-2S (234.95 ±28.23), KY-10S (206.67 ±26.77), and YES-1S (145.55 ± 24.52).

Interestingly, each of the curcumin-surviving cell lines showed a significant decrease in the percentage of cells in the high-staining subpopulation when compared to their corresponding original line (t=3.7235, p= 0.0040). This decline is visible as a loss of nearly all of the cells in the high-staining subpopulations (Figure 5). TE-8 and TE-1 showed complete loss of its high-staining population. The percentage of cells in the high-staining subpopulations of the curcumin-surviving lines ranged from 0 for TE-8S and TE-1S to 12.2% for KY-5S.

### Detection of CD44-expressing cells in ESCC and curcumin-surviving lines

**Figure 6:**
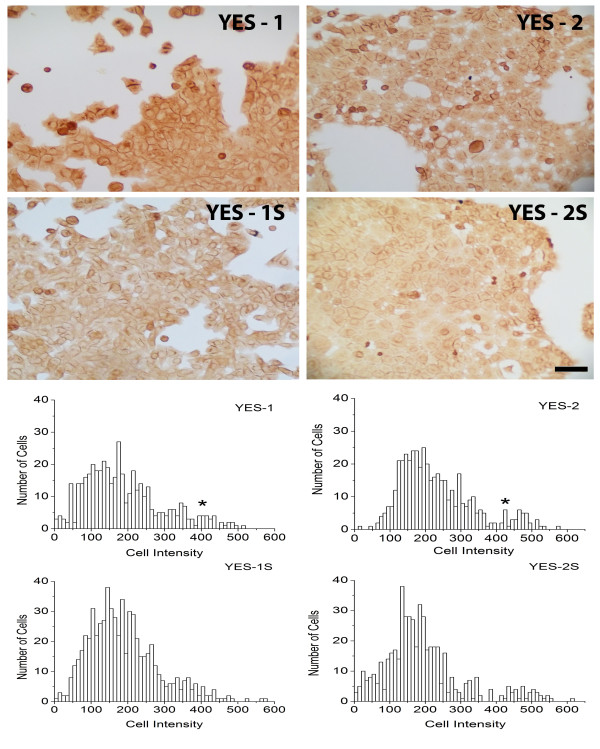
**CD44 immunocytochemistry. **Above: two of the six original ESCC lines (YES-1 and YES-2) and their curcumin-surviving lines (YES-1S and YES-2S) stained with anti-CD44. Below: histograms showing the high-staining subpopulations in the original cell lines (peak marked by asterisk) which is diminished in the surviving lines. Scale bar =100 μm. There were significant high-staining cell numbers within all original and surviving lines. The mean intensity of high-staining cells was 446.47 ± 31.21, 438.96 ± 55.38, 463.79 ± 50.66, and 470.20 ± 53.77 for YES-1, YES-2, YES-1S, and YES-2S, respectively (F=228, p<0.0001).

CD44 expression was examined in two ESCC lines with high ALDH1A1 expression, YES-1 and YES-2, and their corresponding curcumin-surviving lines, YES-1S and YES-2S. As shown in Figure 6, the cells in all four lines were distributed into two significantly different subpopulations according to CD44-staining intensity (F=228, p<0.0001). The mean of the high intensity-staining cells was about twice that of low intensity-staining cells. For example, the mean intensities of the low- and high-staining YES-1 subpopulations were 175.47 ± 89.99 and 446.47 ± 31.21. The mean intensity of the high-staining cells of the other three lines was 438.96 ± 55.38, 463.79 ± 50.66, and 470.20 ± 53.77 for YES-2, YES-1S, and YES-2S, respectively. The percentage of high-staining cells in the original lines was 12.11% and 4.82% for YES-2 and YES-1, respectively. The percentage of high CD44-staining cells decreased in the curcumin-surviving cell lines to 8.67% and 2.70% for YES-2S and YES-1S, respectively.

### Tumorsphere formation

**Figure 7:**
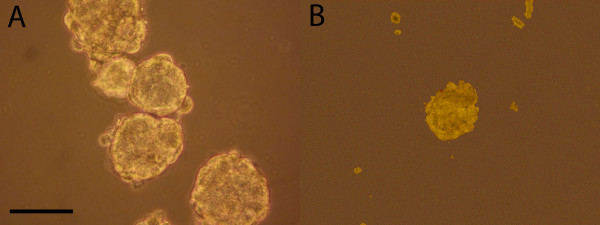
**Tumorsphere formation. **(**A**) Anchorage-independent spheres formed from YES-2 cell line after 7 days in stem cell medium. (**B**) Spheres formed from YES-2S after 2 weeks of cell culture. Scale bar=100 μm.

The ability of the original YES-2 and its surviving line (YES-2S) to form three-dimensional spheres in serum-free medium containing EGF and bFGF on low-attachment plates was examined. This procedure has been reported by others to identify stem cells [59-61]. Under these conditions, YES-2 cells grew as anchorage-independent spheres after 7 days of culture (Figure 7A), whereas YES-2S cells did not form any spheres at that time. The average number of spheres formed by YES-2 was 206 ± 20.8 (mean of all spheres in 3 plates ±SD) and sphere size ranged from 70–200 μm in diameter. However, YES-2S cells were only able to form spheres after two weeks, and the average number of spheres was 4.3 ±5.13 (mean of all spheres in 3 plates ±SD) with diameters ranging from 40–100 μm (Figure 7B). These results indicated that the original line produces greater numbers of spheres that are larger in size and grow faster than the spheres in the curcumin-surviving line.

### Effect of curcumin treatment on the curcumin-surviving lines

**Figure 8:**
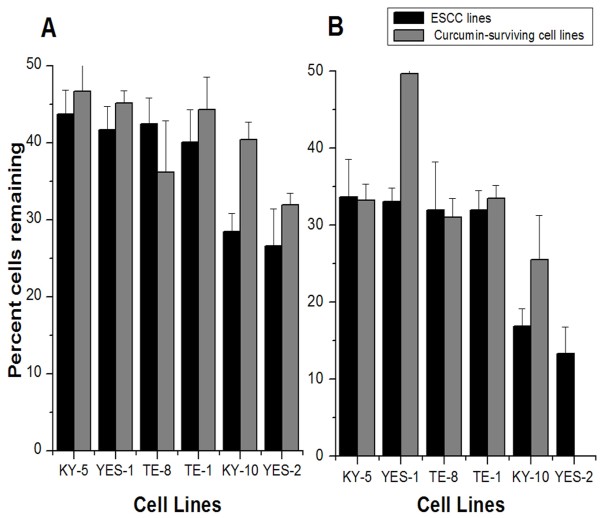
**Effects of curcumin on curcumin-surviving cell lines. **Overall, the surviving lines showed a small but significant increase in survivorship to **A** 40 μM (F=5.132, p=0.031) and **B** 60 μM (F=8.358, p=0.008) curcumin administered for 30 hrs. Data for the original lines are from Figure 2. Cell lines are arranged by responses of the original lines to 60 μM curcumin. The mean of three experiments ± SD is shown for 40 and 60 μM curcumin.

To compare the effect of curcumin on the ESCC lines and curcumin-surviving lines, the curcumin-surviving cell lines were passaged four times and treated with 40 or 60 μM curcumin. The resulting cell density was determined by standard crystal violet assay and the percentage of cells remaining after treatment was calculated for each line (Figure 8). This experiment was repeated three times. A two-way ANOVA revealed a small but significant increase in resistance in the curcumin-surviving cell lines relative to the original lines when tested with either of the two-curcumin concentrations (factor 1; 40 μM: F=5.132, p=0.031; 60 μM: F=8.358, p=0.008; Scheffe post hoc test). YES-1S showed the greatest change from its original cell line. The cell lines also differed significantly from each other in their ability to survive the curcumin treatments (factor 2; 40 μM: F=9.508, p<0.001; 60 μM: F=12.71, p<0.001; Scheffe test).

## Discussion

Esophageal cancer is a highly aggressive malignant disease resulting in low patient survival. Because of the poor success rate of standard therapies, innovative approaches for treatment have been tested, and two studies have examined whether curcumin could be a potential candidate for esophageal cancer treatment. In one study, Hartojo et al. [62] examined the effects of curcumin on the NF-κB activity and cell viability of esophageal adenocarcinoma and also in combination with 5-fluorouracil (5-FU) and cisplatin. In the other study, O’Sullivan-Coyne et al. [63] investigated the effect of curcumin-induced apoptosis on esophageal cancer cells. In the present investigation, we selected six esophageal squamous cell carcinoma lines to evaluate their sensitivity to curcumin. The six cell lines were found to be similar morphologically to what was shown in the original publications introducing these lines [53-56] (Figure 1, Table 1).

When the cell lines were treated with curcumin (20–80 μM) for 30 hrs there was a reduction in cell number in all lines in a dose-dependent manner, consistent with studies using breast, lung, and colorectal cancer cell lines (reviewed by Anand et al. [14]), and esophageal cancer cell lines (OE19, OE33, OE21, KYSE450) [63]. We found a significant difference in the response of the six ESCC lines to curcumin according to the crystal violet assay. The KY-5 cell line, for example, showed about three times more cells remaining after 60 μM curcumin than YES-2 (Figure 2).

This is the first study to characterize curcumin-surviving cell lines. All six curcumin-surviving cell lines had lower levels of ALDH1A1 when compared with each corresponding original line, based on ELISA and immunocytochemistry. ALDH1A1 is a stem cell marker that offers cellular protection against cytotoxic drugs [4,41,64-66]. High ALDH1A1 expression was detected in a subpopulation of candidate CSCs in all six ESCC cell lines, and there was considerable variation among the lines (Figures 4 &5). This is also the first report to analyze ALDH1A1 expression in ESCC lines. Although in theory CSCs are thought to comprise a small percentage of the cancer cell population, the percentage of high ALDH1A1-staining cells that we found (5.6% to 40.4%.) is consistent with the high percentage of CSCs identified in other cancer cell lines [43,67]. Interestingly, the high-expressing subpopulation was diminished or lost in all of the surviving lines, indicating that CSC numbers are altered by curcumin.

Across the six lines, levels of ALDH1A1 and NF-κB were inversely correlated with sensitivity to curcumin, as exemplified by KY-5 versus TE-1 (Figure 3). Because one of the functions of ALDH1A1 and NF-κB is to protect against cytotoxic agents, the higher levels of both proteins found in cell lines with lower sensitivity to curcumin suggests that they could be protecting against curcumin’s effects. Additional studies should examine whether ALDH1A1 does indeed inhibit curcumin toxicity towards cancer cells. Furthermore, the relative ALDH1A1 expression across the lines as determined by anti-ALDH1A1 immunocytochemistry agreed with the ELISA results, as shown in Figures 3–5. Similarly, the two cell lines with high ALDH1A1 and NF-κB levels, KY-5 and YES-1, were the least sensitive to curcumin, whereas the more sensitive lines (KY-10, TE-1, TE-8) had lower expression of these two markers.

This study examined p65, a subunit of the NF-κB transcription factor, in the ESCC lines. The results support use of NF-κB as a marker for cancer stem cells because NF-κB and ALDH1A1 levels were strongly correlated. NF-κB levels decreased among the three surviving cell lines that had the highest initial levels of NF-κB (KY-5, YES-1 and YES-2) according to ELISA. Additionally, the other three lines showed the same (KY-10) or more NF-κB expression (TE-1 & TE-8) in the surviving lines, and this result was confirmed using immunocytochemistry with the same three lines (TE-1, TE-8, KY-10, data not shown). The increase in NF-κB but not ALDH1A1 in two lines could result from the differences between the functions of these markers. For example, ALDH1A1 is an enzyme, whereas NF-κB is a cell signaling molecule and transcription factor that serves in multiple cellular pathways. This difference suggests that ALDH1A1 may be a better indicator of stem-like cells.

Whereas most studies measure the activity of the NF-κB signaling pathway, such as nuclear entry of p65, we determined the level of p65 in the ESCC lines. Several studies have shown that the level of NF-κB in cells is correlated with aggressiveness [68,69], lack of differentiation [70,71], and resistance to chemotherapy [72]. Kang et al. [68] reported that p65 was expressed more strongly and regularly in ESCC tissues than in normal esophageal tissues. Additionally, Tian et al. [69] reported that two ESCC cell lines (Eca109 and EC9706) showed high p65 expression, and the sensitivity of these cell lines to a chemotherapeutic agent increased after p65 expression was inhibited by p65 siRNA transfection. Zhang et al. [70] reported that the expression level of NF-κB was higher in poorly or moderately differentiated lung cancer cells than in well-differentiated cancer cells. Yu et al. [71] also found that the density of p65-positive cells was significantly increased in the transition from normal mucosa to adenoma and to adenocarcinoma in colorectal cells. Hatata et al. [72] reported that a high NF-κB level in two ESCC cell lines was associated with poor sensitivity to 5-fluorouracil. The variation in NF-κB level we found among the six ESCC lines could indicate differences in the degree of aggressiveness and degree of resistance to chemotherapy.

Interestingly, YES-2 differed from the other lines: Although it had high NF-κB and ALDH1A1 expression, it showed more sensitivity to curcumin and was the only line that had no survivors after the 60 μM curcumin treatment. It is not clear why the YES-2 cell line behaved differently, and it should be investigated further. Notably, YES-2 is the only one of the six lines derived from the tumor that was reported to have metastasized in the patient [73].

Expression of CD44, a stem cell marker, was examined in two of the lines, YES-1 and YES-2, and in their curcumin-surviving lines. These original cell lines were chosen because they had high ALDH1A1 and NF-kB expression. As observed with ALDH1A1, two subpopulations of cells were recognized in these lines, one with high and one with low CD44 immunostaining. The intensity was two times greater in the high-staining cells than in the low-staining cells. However, the percentage of highly-stained cells was less then what was observed in the ALDH1A1 experiment. For YES-1 and YES-2 the percentage of highly stained cells was 20% and 36.2% for ALDH1A1, respectively, and 4.82% and 12.11% for CD44. One way to explain why the high-staining CD44 subpopulation was smaller than the high-staining ALDH1A1 subpopulation is that the first group may contain mostly CSCs, whereas the second group may include progenitor cells derived from CSCs, which are not fully differentiated but continue to express substantial amounts of ALDH1A1 [74].

It is generally believed that CSCs are more numerous in tumors after chemo- and radiation therapy because they are more resistant than non-stem cells, which along with their greater aggressiveness makes CSCs a likely source of tumor recurrence [75-77]. In contrast to standard therapies, we found that both the high ALDH1A1 and the CD44 subpopulations were diminished in the curcumin-surviving lines, suggesting that the cell populations surviving curcumin contain fewer stem-like cells. Additional evidence that the surviving lines have reduced numbers of CSCs was provided by the ability of YES-2 cells to form tumorspheres, whereas these were sparse, small, and slow-growing in the YES-2S cultures. These results are in agreement with a recently published study in which curcumin was found to inhibit tumorsphere formation in two esophageal adenocarcinoma cell lines [59]. The investigators attributed this effect to inhibition of CSCs by curcumin.

Multiple studies have demonstrated that invasion and metastasis are mediated by a cellular component that displays high ALDH1 activity [27,78], suggesting that ALDH1A1 can be a significant target for cancer therapy. Therefore, the expression of ALDH1A1 in the curcumin-surviving cell lines was evaluated to test for any effect of curcumin on ALDH1A1 expression that is transmissible through cell division and how this property may differ among the cell lines. Because it appeared that we had selected for cells that were intrinsically more resistant to curcumin, we predicted that the surviving lines would be considerably less sensitive to curcumin than the original lines. Although one surviving cell line (YES-1S) showed substantially greater resistance to curcumin, the overall resistance after having been exposed to curcumin was significant but small when all of the lines were examined (Figure 8).

If the sensitivity of each line to curcumin depends, at least in part, on its ratio of CSCs to non-CSCs, then the loss of CSCs in the surviving cell lines, as shown by the immunostaining for stem cell markers, suggests that curcumin sensitivity decreases as the proportion of CSCs declines. However, when comparing across the original six lines, a decrease in sensitivity to curcumin was correlated with increasing stem-like characteristics, as epitomized by KY-5. One explanation for this apparent contradiction is that the range of sensitivity to curcumin detected across the lines could depend more on genetic differences among the lines than on sensitivity of CSCs to curcumin. In support of this explanation, the surviving lines appear in roughly the same order of sensitivity to curcumin as the original lines, despite having lost most or nearly all of their CSCs, as identified by the number of cells with high ALDH1A1 or CD44 levels.

The specific mechanism causing the reduction in stem cells by curcumin was not examined in this study, but we can consider two possibilities. First, the curcumin may have selectively destroyed the CSCs. Second, curcumin may have caused the CSCs to differentiate more frequently into non-CSCs, similar to what has been described in glioma and PCC4 embryonal carcinoma cells [79,80]. The surviving population would, in either case, contain proportionately fewer CSCs. In agreement with the second scenario, CSCs divide symmetrically, forming new CSCs, as well asymmetrically, generating non-CSCs. Curcumin may have altered the balance between these processes, causing more CSC differentiation. It has been argued that the cell culture environment used with cancer cells can determine the percentage of CSCs present [81]. If the microenvironment of tumors also plays a major role in determining the CSC versus non-CSC composition, then curcumin treatments may improve patient outcome and survivorship by acting on CSCs. The surviving cell lines may have also changed while in cell culture during the few passages before they were tested with the second curcumin treatment. Nevertheless, it is apparent that curcumin reduced the stem cell properties of the lines by either targeting CSCs or by inducing a change in CSCs resulting in cells with reduced stem-like properties. The presence of CSC markers declined and resistance increased in the surviving lines, which agrees with our hypothesis that the CSCs in the surviving lines are reduced in number, because fewer CSCs would be expected to make the ‘S’ line less sensitive to curcumin.

In agreement with both of these possible mechanisms, researchers have shown that curcumin can modulate or eliminate a variety of cellular targets in cancer cells [82,83]. Although curcumin appears to be effective against CSCs, its limited bioavailability in the body may reduce its effectiveness [18,19]. However, Lim et al. [84] reported that nanoparticle-encapsulated curcumin used to treat glioblastoma and medulloblastoma cells did reduce the number of CD133-positive stem-like cells. Also, Kanwar et al. [85] reported that difluorinated-curcumin, a novel curcumin analog, together with other chemotherapeutic agents reduced the CSC cell markers CD44 and CD166 in chemo-resistant colon cancer cells. Our results provide evidence that curcumin alone can reduce the stem-like cell population in ESCC lines.

## Conclusions

The present study demonstrates that curcumin reduces the number of ESCC cells in a dose-dependent manner. By comparing the six ESCC lines the results showed that ESCC cells vary considerably based on CSC properties and sensitivity to curcumin. This study is the first to establish and characterize curcumin-surviving subpopulations among cancer cell lines. By comparing the original ESCC lines and the curcumin–surviving lines we found that the curcumin-surviving cell lines contain fewer stem-like cells than the original lines. These data support clinical applications for curcumin as a chemotherapeutic agent against cancers, particularly ESCC, rather than only as a preventative dietary supplement. Contrary to other chemotherapeutic agents, we predict that the cells that comprise a recurrent esophageal tumor after curcumin treatment would contain fewer CSCs than the original tumor. Because CSCs are considered aggressive and more metastatic, a tumor developing after curcumin treatment may be managed more easily than traditionally treated cancers.

## Abbreviations

ESCC: Esophageal squamous cell carcinoma; CSCs: Cancer stem cells; ALDH1A1: Aldehyde dehydrogenase-1A1; CD44: Cluster of differentiation 44; NF-κB: Nuclear factor kappa-light-chain-enhancer of activated B cells.

## Competing interests

The authors have no conflicts of interest.

## Authors’ contributions

All three authors contributed equally to the research and manuscript preparation. All authors read and approved the final manuscript.

## Pre-publication history

The pre-publication history for this paper can be accessed here:

http://www.biomedcentral.com/1472-6882/12/195/prepub
